# Real-Time Observation of Temperature-Induced Surface Nanofaceting in M-Plane α-Al_2_O_3_

**DOI:** 10.1021/acsami.1c22029

**Published:** 2022-06-28

**Authors:** Denise J. Erb, Jan Perlich, Stephan V. Roth, Ralf Röhlsberger, Kai Schlage

**Affiliations:** † Institute of Ion Beam Physics and Materials Research, Helmholtz-Zentrum Dresden-Rossendorf HZDR, 01328 Dresden, Germany; ‡ Photon Science Department, Deutsches Elektronen-Synchrotron DESY, 22607 Hamburg, Germany; § Institut für Optik und Quantenelektronik, Friedrich-Schiller-Universität Jena, 07743 Jena, Germany; ∥ Helmholtz Institute Jena, 07743 Jena, Germany; ⊥ Helmholtz Centre for Heavy Ion Research GSI, 64291 Darmstadt, Germany

**Keywords:** crystal surface reconstruction, nanofaceted α-Al_2_O_3_, pattern formation, *in situ* grazing incidence small-angle X-ray scattering, GISAXS modeling, atomic force microscopy

## Abstract

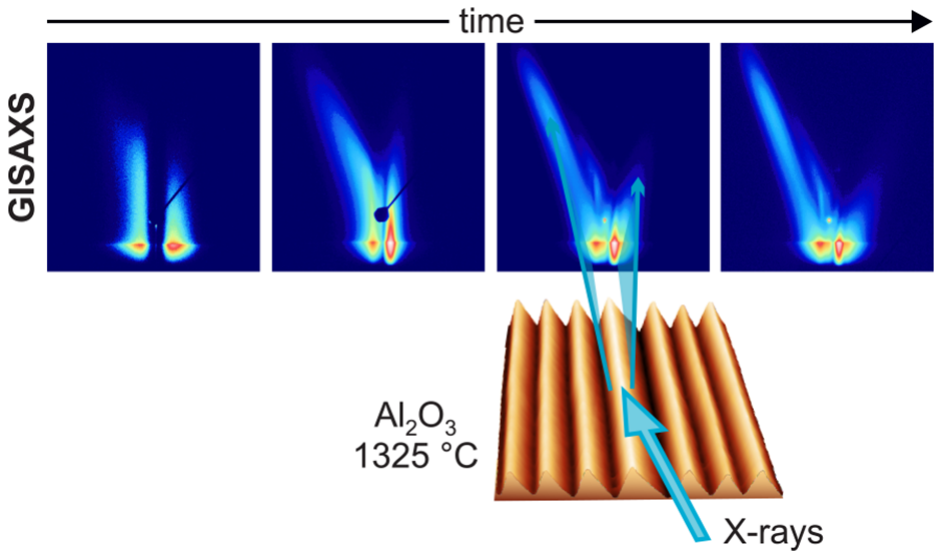

The spontaneous crystal surface reconstruction of M-plane α-Al_2_O_3_ is employed for nanopatterning and nanofabrication in various fields of research including, among others, magnetism, superconductivity, and optoelectronics. In this reconstruction process the crystalline surface transforms from a planar morphology to one with a nanoscale ripple patterning. However, the high sample temperature required to induce surface reconstruction made *in situ* studies of the process seem unfeasible. The kinetics of ripple pattern formation therefore remained uncertain, and thus production of templates for nanofabrication could not advance beyond a trial-and-error stage. We present an approach combining *in situ* real-time grazing incidence small-angle X-ray scattering experiments (GISAXS) with model-based analysis and with *ex situ* atomic force microscopy (AFM) to observe this morphological transition in great detail. Our approach provides time-resolved information about all relevant morphological parameters required to trace the surface topography on the nanometer scale during reconstruction, i.e., the time dependence of the pattern wavelength, the ripple length, width, and height, and thus their facet angles. It offers a comprehensive picture of this process exemplified by a M-plane α-Al_2_O_3_ surface annealed at 1325 °C for 930 min. Fitting the model parameters to the experimental GISAXS data revealed a Johnson–Mehl–Avrami–Kolmogorov type of behavior for the pattern wavelength and a predominantly linear time dependence of the other parameters. In this case the reconstruction resulted in a crystalline surface fully patterned with asymmetric ripple-shaped nanostructures of 75 nm periodicity, 15 nm in height, and 630 nm in length. By elucidating the time dependence of these morphological parameters, this study shows a powerful way to significantly advance the predictability of annealing outcome and thus to efficiently customize nanopatterned α-Al_2_O_3_ templates for improved nanofabrication routines.

## Introduction

Nanopatterned surfaces for application in bottom-up nanofabrication can be obtained from various self-assembly mechanisms, induced for instance by directed material deposition, ion beam irradiation, exposure to chemical reactants, or thermal annealing. A well-known thermally induced self-assembly mechanism is the spontaneous crystal surface reconstruction of α-Al_2_O_3_.^1−5^ Here, a planar α-Al_2_O_3_ surface with M-plane orientation transforms into a nanoscale hill-and-valley morphology with well-defined facet orientations upon high-temperature annealing. These nanopatterned α-Al_2_O_3_ surfaces are widely applied—the most recent examples include the growth of semiconductor nanowires and carbon nanotubes guided by the highly anisotropic surface topography of annealed α-Al_2_O_3_, which has attracted much attention for its ability to produce nanostructures with outstanding mechanical, electrical, and especially optoelectronic properties.^6−13^ The temperature-induced nanopatterning mechanism in α-Al_2_O_3_, first reported by Susnitzky, Heffelfinger, and co-workers,^14−16^ is applied in many more scientific fields: Early works began studying dewetting from α-Al_2_O_3_ and interface interactions of α-Al_2_O_3_ with glasses and metals at high temperatures.^17−22^ Soon after, the uniaxially modulated topography of the faceted α-Al_2_O_3_ surface was used to induce anisotropy in the lateral arrangement and thus in the optical properties of nanoparticle assemblies.^23^ Via physical vapor deposition with geometrical shading, ferromagnetic or superconducting metal nanowires and thin films were grown on faceted α-Al_2_O_3_ and examined with respect to crystal structure, magnetization reversal and magnetic anisotropies, or vortex pinning and guiding.^24−28^ In this regard, we have investigated the development of ferromagnetism and shape-induced magnetic anisotropy *in situ* in a growing Fe thin film on nanorippled α-Al_2_O_3_.^29^ α-Al_2_O_3_ with faceted surface structures has also been applied in nanopatterning and nanofabrication in combination with polymeric materials: A soft polymer replica of the faceted surface can be used as a stamp, or the hill-and-valley topography can be employed to induce long-range lateral chemical ordering in diblock copolymer thin films supported on annealed α-Al_2_O_3_ substrates.^30−33^ Employing the latter approach, we have demonstrated a three-step hierarchical self-assembly process for preparing metallic nanostructure arrays with high degrees of lateral ordering.^34^ With researchers’ interest focusing on applications of nanofaceted α-Al_2_O_3_ surfaces, the actual structure formation processes have rarely been investigated experimentally. Interesting exceptions are the recent studies of step bunching to form periodic patterns on vicinal surfaces.^35,36^ The process of reconstruction of the unstable M-plane surface discussed here offers much flexibility, as the facet sizes and inclination angles change slowly during annealing and could thus, in principle, be chosen specifically to match a given purpose in the optimal way. For example, to successfully induce the lateral alignment of chemical domains in a diblock copolymer thin film on a nanofaceted α-Al_2_O_3_ surface, it is required that the facet width matches the equilibrium domain period of the polymer and that a certain ratio is maintained between the facet height and the polymer film thickness.^31,37^ However, the expected facet dimensions cannot be predicted well without precise knowledge of the patterning kinetics upon annealing. Thus, these applications suffer from having to resort to a trial-and-error approach in preparing the nanorippled α-Al_2_O_3_ surfaces, resulting in a high percentage of rejects and thus low efficiency.

Early studies found that the M-plane {101̅0} surface of α-Al_2_O_3_ has a comparatively high surface free energy density and is therefore metastable.^14,38^ While increasing the surface area, a reconstruction into facets of lower surface free energy density reduces the total surface free energy and is thus predicted to occur spontaneously upon high-temperature annealing when diffusive mass transport on the surface is enabled.^1−5^ The initially planar M-plane {101̅0} α-Al_2_O_3_ surface then reconstructs into a rippled morphology with facets of R-plane {11̅02} and S-plane {101̅1} orientation, where the facet ridges run parallel to the [12̅10] direction.^15,16,30^ The facet ridges have a larger contribution to the surface free energy than a planar surface, so their number is decreased by coarsening of the ripple morphology to further minimize the total surface free energy. At equilibrium, the facets enclose angles of ϑ_*R*_ = 32.4° and ϑ_*S*_ = 17.6° with the initial M-plane surface orientation.

*In situ* studies, necessary to understand the actual kinetics of structure formation especially during the early phase of annealing, seemed to be hardly feasible so far: As the process takes places at high temperatures above 1000 °C and the resulting surface features are nanoscopic in size, a contact-less method with subnanometer resolution is needed. Because the structure formation sets in spontaneously and inhomogeneously on the surface, its *in situ* observation also requires a method that averages over a significant portion of the surface area with adequate speed. To approach these challenges in studying the α-Al_2_O_3_ surface faceting process, we combine an *in situ* real-time grazing incidence small-angle X-ray scattering (GISAXS) observation with modeling and *ex situ* atomic force microscopy (AFM). The reciprocal space information gained from the GISAXS experiment can then be compared with a GISAXS model and with structure formation theory and be related to real space information obtained from *ex situ* AFM. This provides a comprehensive description of the nanopatterning process in M-plane α-Al_2_O_3_, which can advance the predictability of annealing outcome and thus help to efficiently customize nanopatterned α-Al_2_O_3_ templates for improved nanofabrication routines.

## Experimental Section

### Sample Preparation

Polished α-Al_2_O_3_ wafers with M-plane orientation were acquired from CrysTec GmbH, Germany. Their initial root-mean-square roughness was σ_rms_ = 0.20 nm. The wafers were cut into samples of 15 mm × 15 mm such that one pair of opposite sample edges was parallel to [12̅10], i.e., the direction along which the facet edges would form upon annealing. Prior to annealing, the samples were cleaned in an ultrasonic bath of acetone at 50 °C for 15 min. In all cases, the samples were annealed in air. Annealing for *ex situ* investigations was performed in a Borel MO 1800 chamber furnace. For the *in situ* GISAXS investigation, a Carbolite STF16/180 tube furnace with a custom-made sample holder was used, as described below. The sample holder featured a step against which the sample edges with [12̅10] orientation were aligned such that they were approximately parallel to the azimuthal direction of the incident X-ray beam.

### Atomic Force Microscopy (AFM)

A Bruker Multimode 8 atomic force microscope was used in tapping mode under ambient conditions to obtain all AFM topography micrographs shown in this paper, employing BudgetSensors Tap190Al-G AFM tips with a nominal curvature radius of 10 nm. The micrographs had a resolution of 512 pixels per 5 μm and were recorded at a scan speed of 1 line/s. The data were processed, plotted, and analyzed by using the software package Gwyddion.^39^

### Grazing Incidence Small-Angle X-ray Scattering (GISAXS)

**Figure 1:**
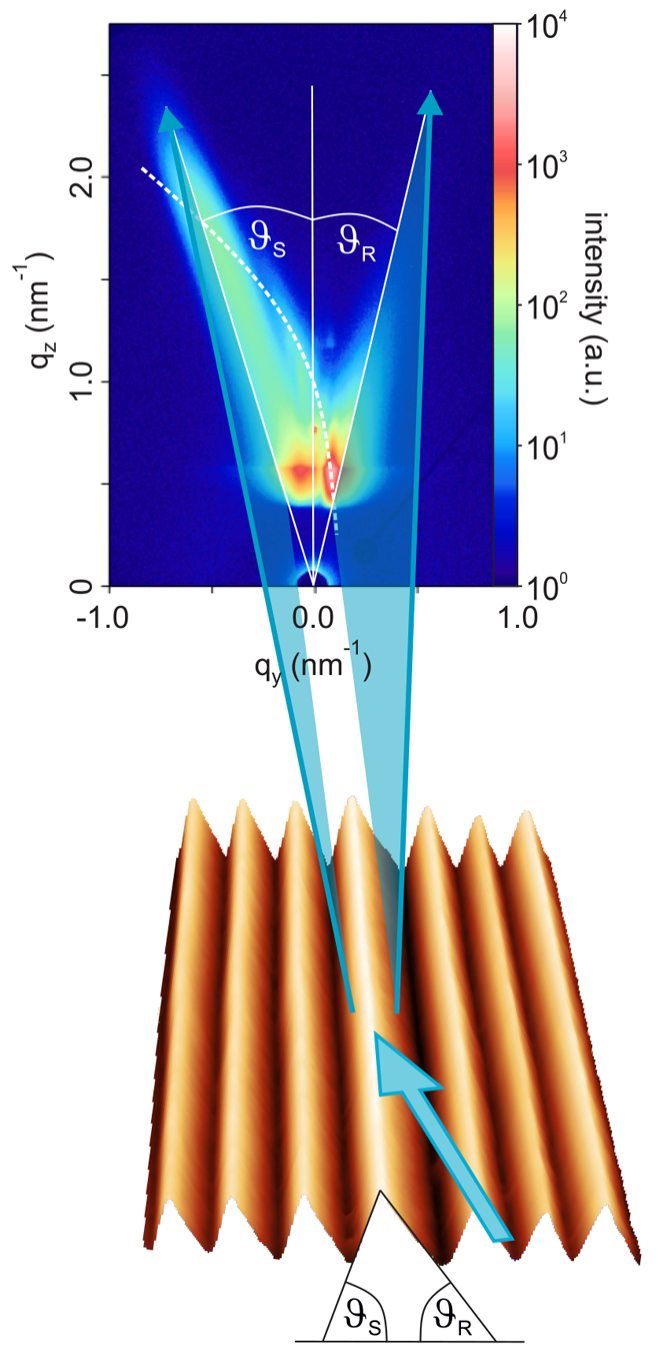
Schematics of the X-ray scattering geometry. The GISAXS intensity map represents a nonequilibrium state, where ϑ_*R*_ < ϑ_*S*_ (see main text for details). Straight white lines represent the vertical specular scattering rod and the tilted facet truncation rods. The dashed line indicates the grating truncation rod.

For *in situ* GISAXS during high-temperature annealing, the tube furnace was set up at the beamline BW4 of the DORIS-III synchrotron^40^ such that the X-ray beam could pass through the tube in grazing incidence geometry (see Supporting Information page S-2f and photographs of the setup). A two-axis goniometer allowed for compensating any sample tilt perpendicular to the X-ray incidence direction and for setting the polar angle of incidence α_i_ of the X-rays. A flight tube was installed to evacuate the sample-to-detector distance of 1840 mm and thus reduce scattering in air. The beam size was 400 μm × 400 μm. A sample was placed onto a custom-made ceramic support at the edge of the furnace tube, and then the support was pushed into the middle of the tube. The ceramic support held the sample in the center of the furnace tube and aligned the sample edges in [12̅10] orientation approximately parallel to the azimuthal direction of the incident X-ray beam. Viewing the sample along the incident X-ray beam direction, the facets with R-plane (S-plane) orientation were on the right (left) hand side of each ripple (see Figure 1).

The sample was then heated at a rate of 7 K/min up to a temperature of 1325 °C and annealed at this temperature for 720 min, with the temperature being regulated to a precision of 1 K by a built-in controller in the furnace. During the heating phase, thermal expansion was compensated by regularly readjusting the sample *z*-position and the X-ray incidence angle by about 50 μm and 0.2°, respectively, in between recording frames such as to maximize the off-specular intensity. GISAXS intensity maps were recorded at an X-ray energy of *E* = 8984.4 eV and an incidence angle of α_*i*_ = 0.5° by using a MAR SX-165 detector with a pixel size of 80 μm. Depending on the intensity scattered to off-specular directions, the exposure time was varied between 60 and 300 s. A round metal plate held by a wire is used as a beam stop to block the intense specular reflection and avoid damage to the detector. The beam stop was required at early times to protect the detector from the intense specular reflection, but could be removed at later times, when the specular reflection had become less intense.

The GISAXS intensity map of the rippled surface is characterized by two tilted facet truncation rods (FTRs) and a curved feature, as indicated by straight and curved lines, respectively, in Figure 1. The FTRs originate from the facet surfaces on each ripple, with the rod tilt angle directly corresponding to the inclination angles ϑ_*R*_ and ϑ_*S*_ of the facet surfaces.^41^ Owing to its resemblance to a grating, a rippled surface also produces grating truncation rods (GTRs).^42^ In case the grating lines or ripples are perfectly parallel to the azimuthal direction of the incident X-ray beam, the intersections of the GTRs with the Ewald sphere are located on a semicircle at the positions given by
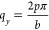
1a
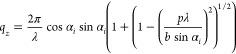
1bwhere *b* is the grating period, λ is the X-ray wavelength, and *p* is the scattering order. As will be discussed below, the sample was rotated azimuthally by ∼2.2° in this experiment. Such a small azimuthal sample rotation φ has a negligible influence on the tilt of the FTRs but drastically alters the locations of the intersections of the GTRs with the Ewald sphere according to
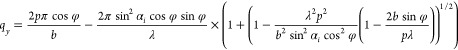
2a
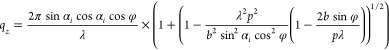
2bso that they are positioned on an elliptic portion. Consider the example discussed by Yan et al. with α_*i*_ = 0.407°, *b* = 450 nm, and λ = 0.1127 nm. For an azimuthal rotation of φ = 1°, this moves the intensity maxima of scattering orders *p* = +5 and *p* = −5 from *q*_*z*_^+,–^ = 0.076 Å^–1^ to *q*_*z*_^+^ = 0.092 Å^–1^ and *q*_*z*_^–^ = 0.044 Å^–1^, respectively, i.e., the effect of azimuthal sample rotation on the GISAXS intensity map of a grating-like surface is indeed significant (see also Figures 7 and 8 of the paper by Yan and Gibaud^42^). Because of the large variation of the ripple width in this sample, the intensity maxima at these intersections were very broadened and not visible individually. They instead showed up as an off-centered curved feature intersecting with the FTR of the left-hand facet surfaces.

### Modeling of GISAXS Intensity Maps

GISAXS intensity maps were calculated based on a model sample surface by using version v1.15.0 (2019-02-25) of the BornAgain software.^43^ A sample was defined to consist of an α-Al_2_O_3_ substrate supporting asymmetric ripple-shaped α-Al_2_O_3_ objects^44^ (see Figure 4a for a sketch of such an object) laterally arranged as a radial paracrystal with size spacing coupling, i.e., the distribution of the distance between two ripples depending linearly on their size. Several model parameters concerning the instrumental setup and fundamental sample characteristics were fixed as listed in Table 1. The refractive index of the sample material was slightly reduced in comparison to the room temperature value to account for the decrease in the density of α-Al_2_O_3_ at the high temperatures used in the experiment.^45^ Parameters that were varied to match the calculated GISAXS intensity maps to the experimental ones in different stages of structure formation are given in the Supporting Information, pages S-4ff. Definitions of all parameters can be found in the publicly available software documentation.^46^

**Table 1:** Parameters Used for Modeling GISAXS Intensity Maps with Version V1.15.0 (2019-02-25) of the BornAgain Software^43^ a

category	parameter	value
instrument	intensity	1 × 10^9^ arb units
	X-ray wavelength	0.138 nm
	inclination angle	0.5°
	detector	rectangular
	alignment	⊥ to direct beam
	resolution function	none
	*X*-axis bins	980
	*X*-axis width	78.2 mm
	*Y*-axis bins	1200
	*Y*-axis width	90.0 mm
	direct beam *u*_0_	39.15 mm
	sample-to-detector distance	1840 mm
	background	constant
refractive index	δ	9.8 × 10^–6^
	β	9.2 × 10^–8^
interference function	function type	radial paracrystal
	damping length	2.5 × 10^5^ nm
	domain size	1000 nm
	size space coupling	1
	probability distribution function	Cauchy 1D
particle layout	approximation	size spacing coupling
	weight	1.000
particle	form factor	ripple2
	*X*, *Y*, *Z* positions	0 nm, 0 nm, 0 nm
substrate	number of slices	1
	top roughness	no

a These parameters were fixed for all cases.

The most relevant parameters in this model are the peak distance of the ripple-shaped objects in the radial paracrystal arrangement (identified with the pattern wavelength λ_*y*_) as well as the ripple length *l* and the ripple width *w*, height *h* and asymmetry length *d*, which determine the facet angles via trigonometric relations. The built-in fitting functionality of BornAgain was used to fit the calculated intensity maps to the experimentally observed ones with the settings detailed in the Supporting Information, page S-8, Table S4.^46^ The fitting was limited to a range of ±50% of the parameter starting value. Not all parameters could be fitted equally well; in case fitting did not produce a satisfying agreement of calculated and experimental intensity maps, the respective parameters were extrapolated and optimized manually. The errors given in the list of parameters in the Supporting Information and in the figures in the main paper are those obtained from the fitting procedure; values without errors were not fitted but adjusted manually.

## Results and Discussion

### Experimental Observations by *Ex Situ* AFM

**Figure 2:**
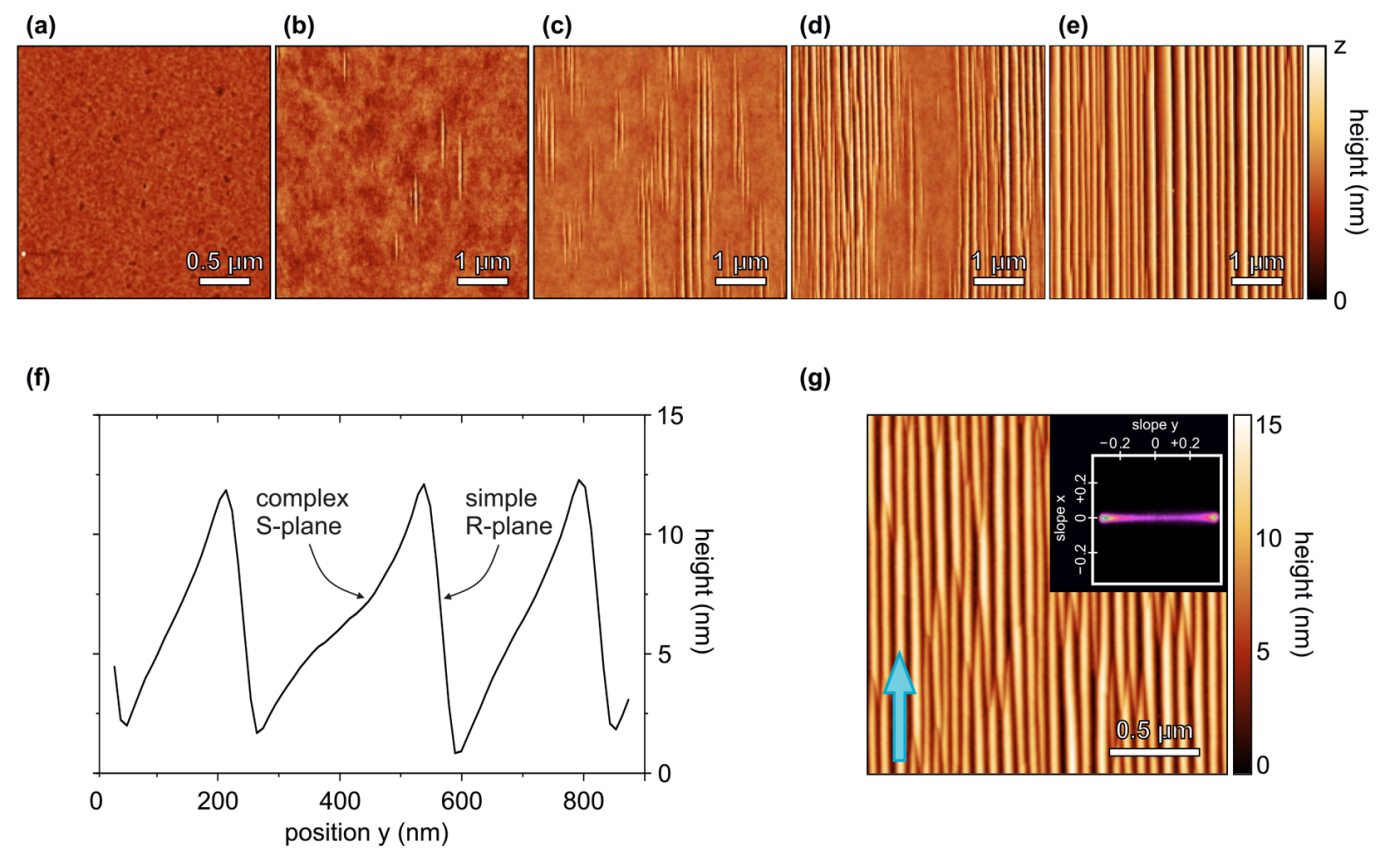
(a–e) *Ex situ* AFM topography images illustrating the successive phases of surface reconstruction in M-plane α-Al_2_O_3_ upon high-temperature annealing for 120 min at 1400 °C. The color bar indicates the depicted maximum height *z*. (a) Initial surface, *z* = 5 nm; (b) formation of individual ripple structures, *z* = 2.0 nm; (c) formation of groups of faceted ripple structures, *z* = 4.5 nm; (d) coalescence of ripple groups, *z* = 5.5 nm; (e) coarsening, *z* = 13 nm. (f) Horizontal line profile of (e), showing the curved S-plane surface on the left and the straight R-plane surface on the right of each ripple. (g) AFM topography image of the sample surface investigated by *in situ* GISAXS in the reconstructed state after annealing for 930 min at 1325 °C. The arrow indicates the azimuthal X-ray beam direction during the *in situ* GISAXS experiment. The inset shows the two-dimensional slope histogram of the surface.

Different stages of the morphological development of the α-Al_2_O_3_ surface after annealing at 1400 °C for 120 min, i.e., the initial phase of surface reconstruction, were identified by *ex situ* AFM as shown in Figure 2a–e, depicting sample areas of 5 μm × 5 μm. We find our results to be in very good qualitative agreement with the results of Heffelfinger and Carter,^16^ who first observed that the reconstruction proceeds in five stages: (1) surface smoothing, (2) formation and growth of individual ripple-shaped, faceted structures, (3) formation of groups of faceted ripple structures, (4) coalescence of ripple groups, and (5) coarsening. The initial sample surface (Figure 2a) was planar with a root-mean-square roughness of σ_rms_ = 0.20 nm. In agreement with the observation of Heffelfinger and Carter,^16^ slight surface smoothing occurred in stage 1, resulting in σ_rms_ = 0.12 nm for the surface in Figure 2b when excluding the emerging ripple structures. This smoothing was not as pronounced as observed by Heffelfinger and Carter, which is explained by the fact that the initial roughness of the sample investigated here was by an order of magnitude smaller than that of the sample studied by those authors. Stage 2 is characterized by the nucleation of isolated faceted, ripple-like structures on the otherwise planar surface (Figure 2b). Figure 2c shows that ripples successively nucleated from the surface height modulations caused by the first ripples; thus, groups of faceted structures formed in stage 3. These groups extended and coalesced in stage 4 (Figure 2d). Junctions were formed because the ridges formed by intersecting facets were generally not aligned among different groups of surface structures. In stage 5 the hill-and-valley morphology coarsened; i.e., ripples grew in height and width at the expense of other, smaller ripples (Figure 2e), driven by reducing the surface free energy via reduction of the number of surface edges formed by facet intersections.^4,5^ In agreement with earlier works,^15,16,25^ we find a straight (“simple”) and a curved (“complex”) ripple surface in *ex situ* AFM (see Figure 2f). In equilibrium, the straight facet surface is attributed R-plane {11̅02} orientation with ϑ_*R*_ = 32.4° and the curved facet surface is attributed approximate S-plane {101̅1} orientation with ϑ_*S*_ = 17.6°.^24^ This equilibrium state was not fully reached in the *in situ* experiment. The surface of the sample used in the *in situ* experiment (depicted in Figure 2g) exhibited ripples with polar facet inclination angles of 16.5° and 19°, corresponding to the peaks at −0.30 and 0.34, respectively, in the slope histogram inset in Figure 2g.

Note that the AFM topography micrographs shown in Figure 2b–e were all recorded at different locations on the same sample after 120 min of annealing at 1400 °C. The initial formation of individual ripples in stage 1 is expected to occur by ripples nucleating at pre-existing localized height modulations. Thus, nucleation is more likely in areas where these modulations are more numerous or more pronounced. Given the inherent inhomogeneity of a real surface, some surface areas of a sample may still be mostly planar, while others may already have developed a pronounced ripple pattern after the same annealing duration. One must therefore be cautious when trying to correlate the results of *ex situ* local-probe techniques such as AFM with the annealing duration. In contrast, the *in situ* GISAXS data detailed below represent the average surface morphology of a sample area of ∼6 mm^2^, the area of the X-ray beam projected onto the sample surface at grazing incidence.

### Experimental Observations by *In Situ* GISAXS

**Figure 3:**
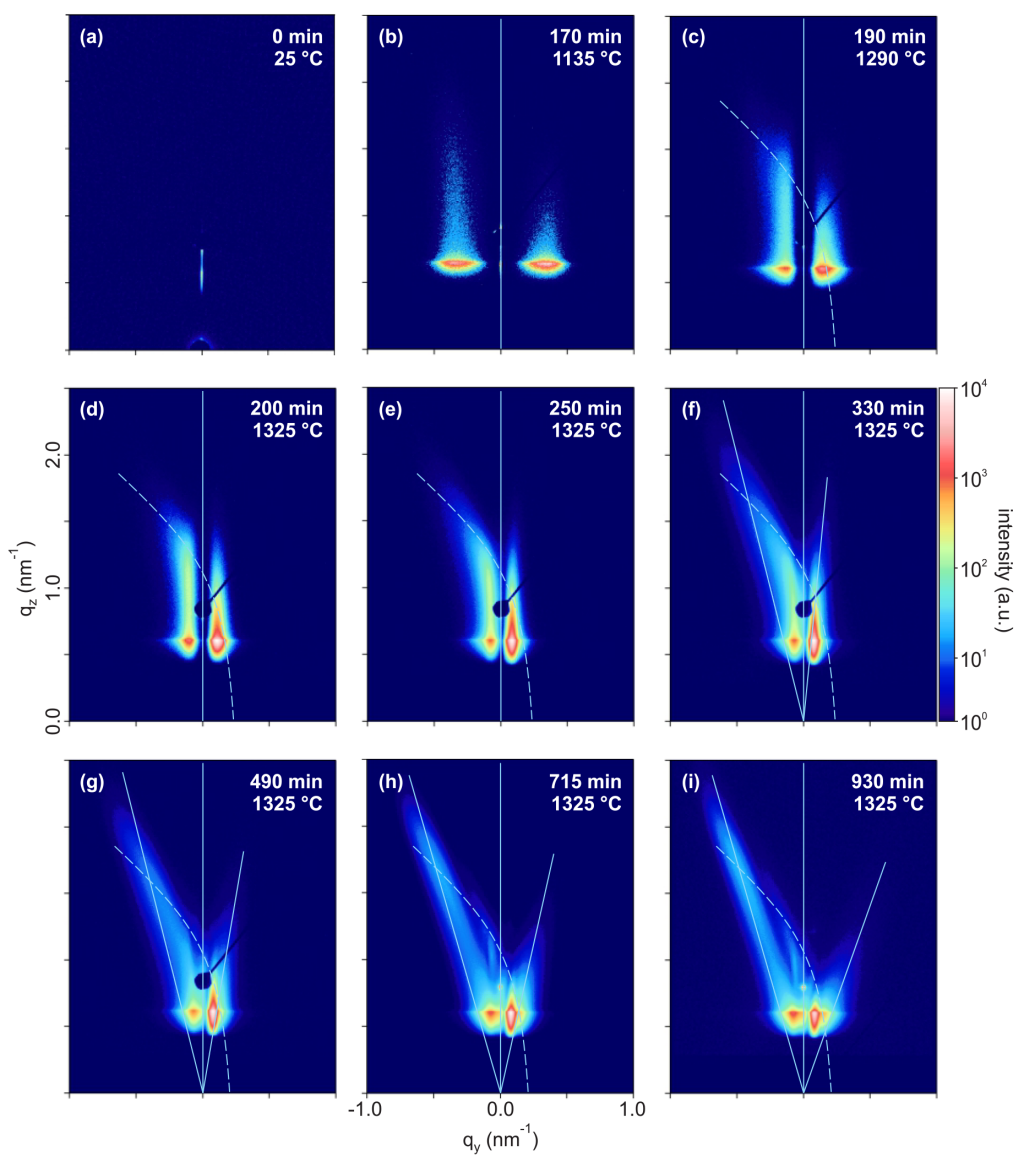
(a–i) Sequence of GISAXS intensity maps recorded during heating and annealing of a M-plane α-Al_2_O_3_ crystal surface. Labels state the respective momentary temperatures and the time elapsed since heating was started. The X-ray energy is *E* = 8984.4 eV, and the angle of incidence is α_i_ = 0.5°. The color bar indicates the scattered intensity in arbitrary units on a logarithmic scale. In (b–i), the vertical lines show the specular scattering plane, the tilted lines denote the orientations of the facet truncation rods, and the curved lines indicate the grating truncation rods. The sample is rotated azimuthally by ∼2.2° off the X-ray incidence direction, which leads to the curved shape of the grating truncation rods.

A sequence of GISAXS intensity maps is shown in Figure 3, with the labels stating the respective momentary temperatures and the time elapsed since heating was started. Relevant morphological characteristics of the reconstructing sample surface can be inferred from these data: The GISAXS pattern of the initially planar sample featured only a specular scattering rod resulting from the initial uncorrelated roughness (Figure 3a). The GISAXS data begin to show significant additional off-specular intensity in the Yoneda region as first indication of a correlated structure width after 155 min, when a sample temperature of 1040 °C, i.e., ∼50% of the bulk melting temperature, was reached. The off-specular maxima then moved to smaller values of *q*_*y*_ and increased in intensity. An asymmetry of the scattering pattern at higher angles α_*f*_ was observable from 170 min and 1135 °C onward (Figure 3b). Starting after 190 min, at a temperature of 1290 °C, tilted rodlike features appeared first on the left and then on the right-hand side of the GISAXS intensity map (Figure 3c,e) and became more pronounced at later times. The tilt of both features changed during annealing (Figure 3c–i), with a more pronounced change on the right-hand side. The occurrence of tilted facet truncation rods is related to the formation of surface structures with well-defined facet inclinations ϑ_*R*,*S*_.^41,47−49^ It is noted, however, that at least the left-hand feature did obviously not intersect with the reciprocal space origin in the early and intermediate stages of annealing. Therefore, this feature cannot be interpreted as a truncation rod of an inclined facet in a straightforward manner,^41^ as will be discussed below.

Once the composition of the GISAXS intensity map is understood, one can relate its changes to the progression of the morphological surface reconstruction caused by annealing, as observed in snapshots by *ex situ* AFM in the work by Heffelfinger and Carter.^16^ As mentioned above, the *ex situ* AFM data in the present work (see Figure 2) were all obtained from one sample after a single annealing step. This is to show that all stages can be present simultaneously in one sample due to inhomogeneous nucleation of the surface reconstruction process. With a beam footprint of 6 mm^2^, the GISAXS measurement is therefore likely to average over surface regions which, at a given time, have reached different stages of reconstruction. As a result, the obtained GISAXS data show a superposition of features characteristic for these different stages, e.g., changes in the position of off-specular maxima (related to the pattern wavelength) and their relative intensity (related to the ripple surface coverage) are observed simultaneously. We can therefore in general not pinpoint the beginning and end of individual reconstruction stages to specific times, but rather observe gradual transitions from one stage to the next, as described in the following.

The experimental GISAXS intensity map taken prior to annealing featured only a specular scattering rod; i.e., the surface roughness was not significantly correlated. During the heating phase before the target annealing temperature was reached, broad and weak vertical off-specular scattering rods became first detectable at high *q*_*y*_ values, corresponding to very small pattern wavelengths λ_*y*_ of a few nanometers, as first ripples and ripple groups formed in stages 2 and 3 as termed by Heffelfinger and Carter,^16^ and then moved to lower *q*_*y*_ values. The off-specular scattering rods became more intense as an increasing area fraction of the sample was covered with ripples. This corresponded to Heffelfinger and Carter’s stage 4, i.e., growth and coalescence of facet groups. In this process, an increasing area fraction of the surface was covered by faceted ripples, and the fraction of planar, horizontal surface area decreased. As the latter area fraction was diminished, so was its contribution to the intensity in the specular scattering rod. As the morphological reconstruction proceeded toward complete faceting of the entire surface, the specular scattering rod became so weak in intensity that the beam stop could be removed. Stage 5, i.e., facet coarsening, ended when the off-specular scattering rods stopped changing their position. and the pattern wavelength saturated after about 400 min. From then on, only the two facet inclination angles continued to increase approaching their equilibrium values as can be seen from the increasing tilt angles of the FTRs. As will be discussed below, the inclination of the S-plane ripple surface developed at a different rate than that of the R-plane surface.

### Modeled GISAXS Intensity Maps

**Figure 4:**
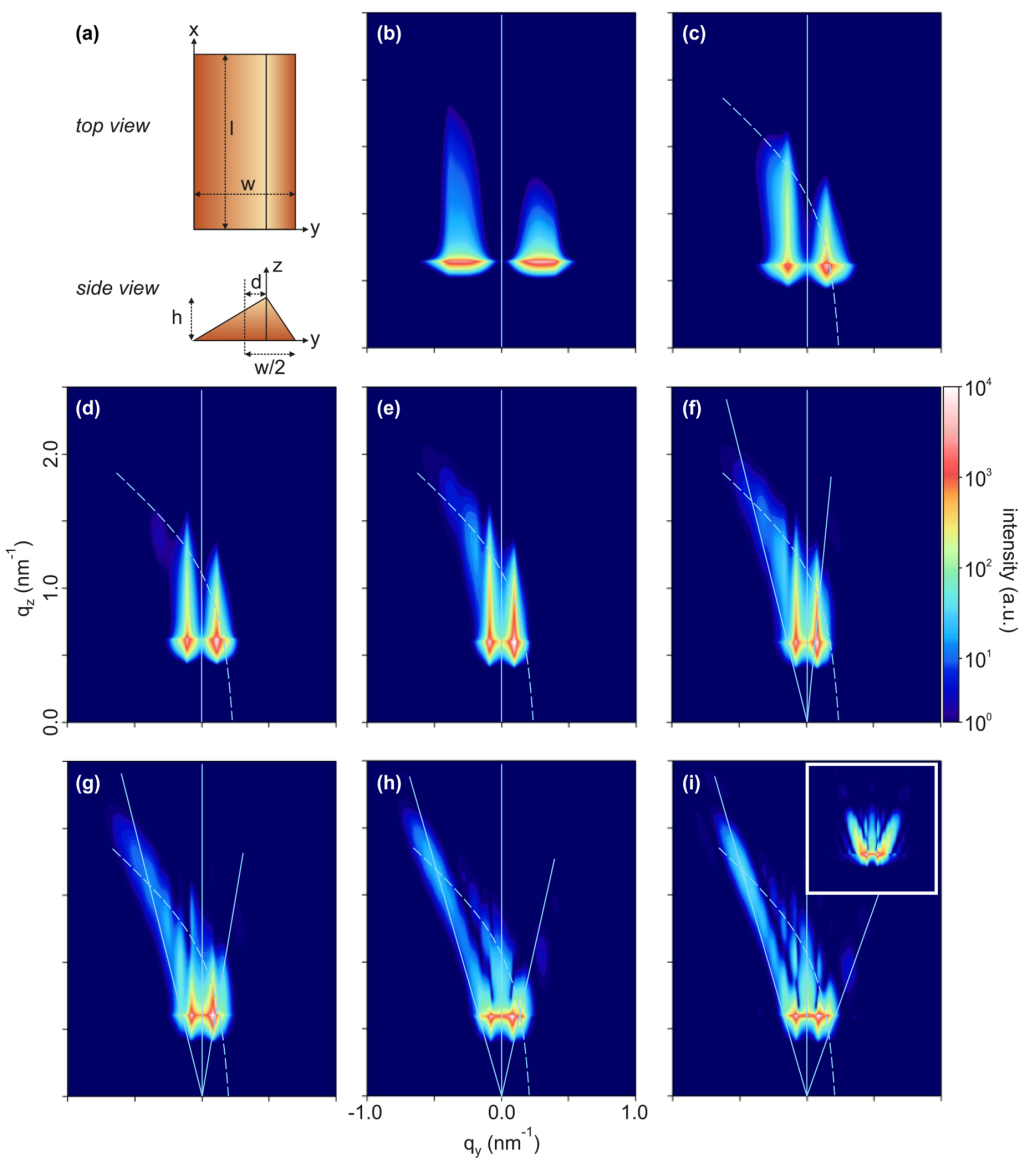
(a) Sketch of the ripple-shaped object described by the form factor used for modeling the GISAXS data. (b–i) Calculated GISAXS intensity maps for subsequent stages of surface reconstruction during annealing of M-plane α-Al_2_O_3_, corresponding to the experimental data shown in Figure 3. The color bar indicates the scattered intensity in arbitrary units on a logarithmic scale. The vertical lines show the specular scattering plane, the tilted lines denote the orientations of the truncation rods originating from the faceted surface, and the curved lines indicate the grating truncation rods. The modeled sample is rotated azimuthally off the X-ray incidence direction by angles as given in Table S1, except in the inset in (i) where the sample is not rotated.

Figure 4 shows a sequence of calculated GISAXS scattering patterns, corresponding to the experimental data in Figure 3. A sketch of the ripple geometry used in the model is shown in Figure 4a; a detailed list of parameters is given in Table 1 and Tables S1–S3. While parameters describing static conditions (such as instrument properties, refractive index, or interference function) were unchanged, we varied the dimensions of the ripples and specifics of their lateral arrangement. Varying the different model parameters allowed to separate the contributions of the superimposed grating truncation rod (GTR) and facet truncation rods (FTR) and to judge the effect of the azimuthal angle on the GISAXS intensity map. For illustration of this effect, a video of experimental GISAXS data taken during azimuthal sample rotation is provided as Supporting Information. When assuming that the azimuthal direction of the incident X-rays is exactly parallel to the ripple ridges, no acceptable agreement with experiment could be achieved. It was necessary to assume an azimuthal incidence angle of ∼2.2° (see Table S1 for the exact values in the individual stages; the variation is attributed to alignment limitations of the high-temperature setup in particular during the heating phase) to obtain a reasonable agreement, as can be seen from Figure 4i: The inset GISAXS intensity map was calculated for the same model parameters as the main subfigure, but with an azimuthal incidence angle of 0°. It does not agree with the asymmetrical scattering pattern observed experimentally (see also the Supporting Information, page S-9, Figure S2, for a comparison of simulated GISAXS intensity maps with different azimuthal incidence angles for exemplary stages of the reconstruction process). The imperfect azimuthal sample alignment did not compromise the interpretation of the data, however, but could be deterministically included in the model via the azimuthal incidence angle.

The overall behavior observed experimentally was then reproduced well with the model, showing first coarsening of the pattern wavelength and later the appearance of rodlike features with increasing tilt angles. Also, an intensity maximum becomes visible at the intersection of the tilted FTRs and the bent GTR. The quantitative information about the kinetics of surface reconstruction obtained from the model will be discussed in the following section.

### Discussion

**Figure 5:**
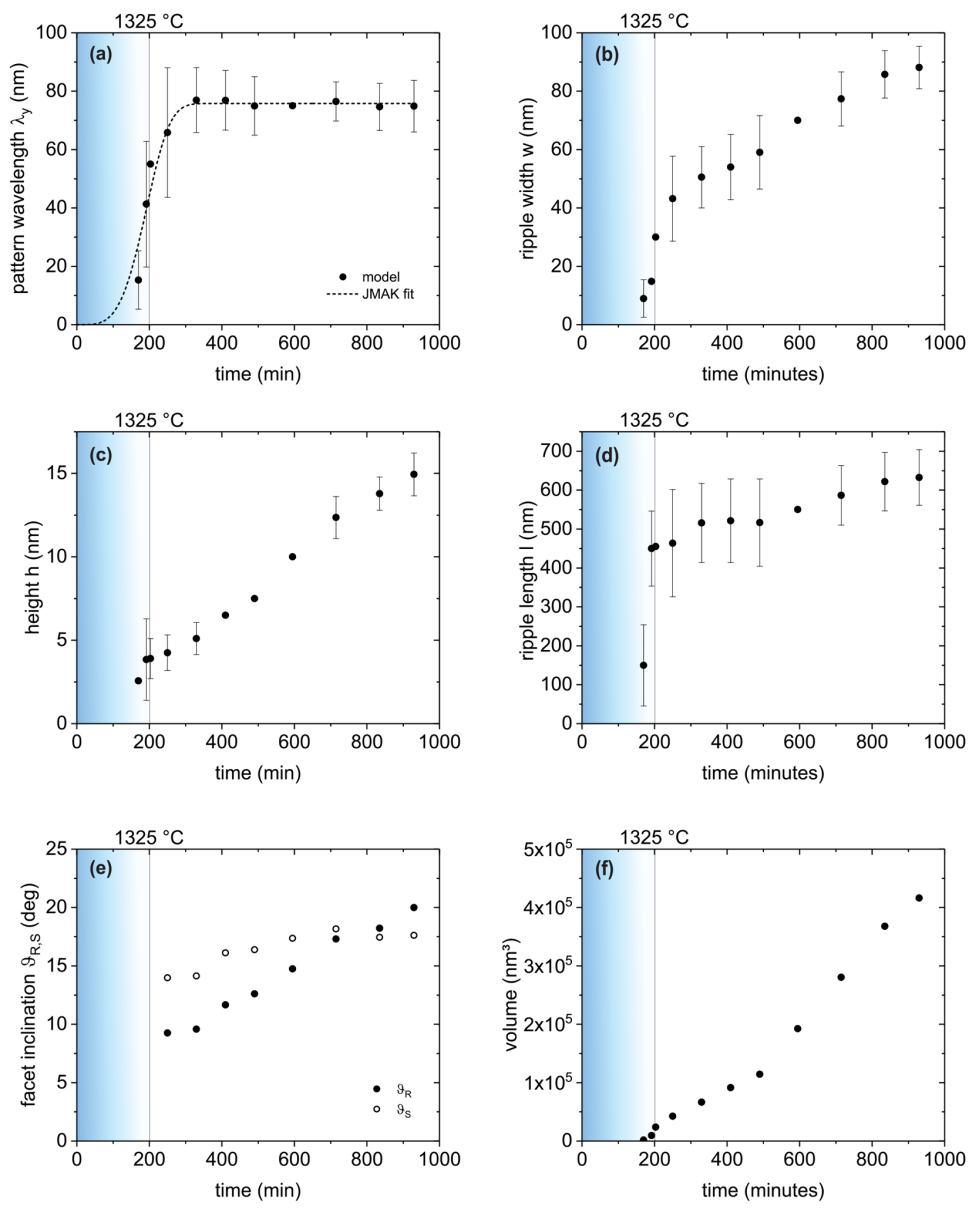
Temporal evolution of the model parameters describing the surface morphology during heat-induced ripple formation: (a) the pattern wavelength, (b) the ripple width, (c) the ripple height, (d) the ripple length, (e) the facet inclinations, and (f) the ripple volume. The color gradient regions mark the heating phase, where the sample temperature increases approximately linearly from room temperature to *T* = 1325 °C.

Evaluating the GISAXS data obtained in this specific experiment was demanding mainly due to two factors: First, the sample surface was significantly inhomogeneous; i.e., different patterning stages were simultaneously present on the surface, and the ripple sizes varied widely (see Figure 2). Second, during annealing the surface morphology changed drastically on a time scale of minutes (see Figure 3). Still, the agreement of the modeled intensity maps with the experimental ones throughout the different stages of the reconstruction process validates the general assumptions of the model. Given the experimental circumstances above, it is merely feasible to obtain averaged morphological parameters with considerable uncertainty. However, the *in situ* concept of the experiment allows for obtaining characteristic kinetics of the surface reconstruction process. The temporal evolution of model parameters describing the morphological changes during crystal surface reconstruction is plotted in Figure 5. The vertical lines at *t* = 200 min indicate the end of the heating phase, when a constant temperature of 1325 °C was reached. In Figure 5a–d values plotted with error bars were obtained from fitting, and those without error bars were extrapolated. In these cases, the fitting procedure found minima for parameters that did not reproduce the prominent features of the experimental GISAXS intensity maps but still resulted in a reduction of χ^2^. The facet inclination angles shown in Figure 5e were calculated from the ripple height, width, and asymmetry length. Because the asymmetry length could not be fitted for the above reason, the angle values are shown without error bars. The errors of the particle volume are not visible on the scale of the plot in Figure 5f. For the pattern wavelength λ_*y*_ (identified with the peak distance of the paracrystal arrangement in the model) we observe first a rapid increase and then saturation at ∼75 nm for *t* > 400 min. The ripple width *w* appears to increase continuously during the observed time span and to finally exceed the pattern wavelength by about 10%. Given that in the model the position of the intense maxima in the Yoneda region is predominantly determined by the value of λ_*y*_, we assume that this further increase of *w* is an overestimation enabled by the less pronounced effect of the ripple width on the scattering pattern. The ripple width should in fact saturate when it equals the pattern wavelength. Assuming a ripple width of *w* = 75 nm for *t* > 650 min and fitting the ripple height again result in a slight increase of the resulting facet inclinations in this time range by up to 1.3°, which is considered a tolerable error. In the observed time span the ripple height *h* increases approximately linearly at a rate of 0.02 nm/min. A saturation at *h* ≈ 18.5 nm is expected when the ripple width saturates and the facet angles reach their equilibrium values of ϑ_*S*_ = 17.6° and ϑ_*R*_ = 32.4°. We find that the facet inclination angles developed at very different rates: The equilibrium value of the S-plane facet was reached after about 600 min, while the inclination of the R-plane facet increased much slower. From the observed rate of 0.018°/min we can extrapolate that the equilibrium value would be reached after ∼1600 min of annealing. The ripple length *l* increases sharply for early times *t* < 200 min and then much slower at a rate of 0.25 nm/min in an approximately linear fashion. This change in rate may be associated with the coalescence of ripple groups in stage 4. Given that increasing the ripple length reduces the surface free energy, a saturation of *l* is not necessarily expected. The volume of an average ripple (calculated from height, width, and length) increases continuously but at varying rates. Another rate change can be expected when the height saturates, and the volume can only increase further by an increase of the ripple length.

The early saturation of the pattern wavelength λ_*y*_ indicates that stages 1 (nucleation) to 5 (coarsening) according to Heffelfinger and Carter^16^ were already completed after ∼400 min, and the surface transformation then further progressed toward the equilibrium state via increase of the facet inclination angles and ripple height. We attempt to empirically describe the dependence of the pattern wavelength on the annealing duration based on the Johnson–Mehl–Avrami–Kolmogorov (JMAK) equation *f*(*t*) = 1 – e^–(*kt*)^*n*^^. This equation can be used for describing the transformed fraction *f* with time *t* in diffusion-controlled structural transformations of homogeneous systems subject to isothermal annealing. The exponent is *n* = 4 in case the transformation proceeds via random nucleation and linear growth in three dimensions. *k* is a dimensionless temperature-dependent factor, resulting from the rates of nucleation and growth.^50,51^ Because we are not considering the kinetics of volume fractions, but that of a non-normalized parameter describing the surface morphology, we use the following modified equation:
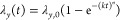
3The dashed line in Figure 5a is a fit to the experimental data according to eq 3 with *n* set to 4, λ_*y*,0_ = 75.8 ± 1.9, and *k* = 0.00484 ± 0.00014. Because the surface reconstruction is a diffusion-driven process, it appears reasonable that the fit is an overestimate for *t* < 200 min, where the actual sample temperature is lower than the final annealing temperature.

Heffelfinger and Carter^16^ investigated α-Al_2_O_3_ {101̅0} surfaces by means of *ex situ* AFM after different annealing durations and observed λ_*y*_ to increase rapidly and then obtain a nearly constant value. They proposed the power law λ_*c*_(*t*) ∝ *t*^0.13^ to model this behavior. Considering the small number of available data points and the large variation of λ_*y*_ in the experiments by Heffelfinger and Carter, this assumption may have been justified then. However, a power law could not fit the temporal evolution of λ_*y*_ obtained from *in situ* GISAXS and presented in this work (see the Supporting Information, pages S-10f, for a comparison of fits according to a power law and eq 3 for the data in this work and for those presented by Heffelfinger and Carter). A power law would not follow the quick increase and abrupt onset of saturation of λ_*y*_, even if only data for *t* > 200 min are considered. While a JMAK type of equation yields a better fit than a power law, we do not claim that the surface reconstruction of α-Al_2_O_3_ is in fact a transformation process such as defined above, for which the JMAK equation was derived. The physical reasons explaining why a JMAK type of equation is a suitable description of the time dependence of the pattern wavelength in α-Al_2_O_3_ reconstruction remain to be clarified.

**Figure 6:**
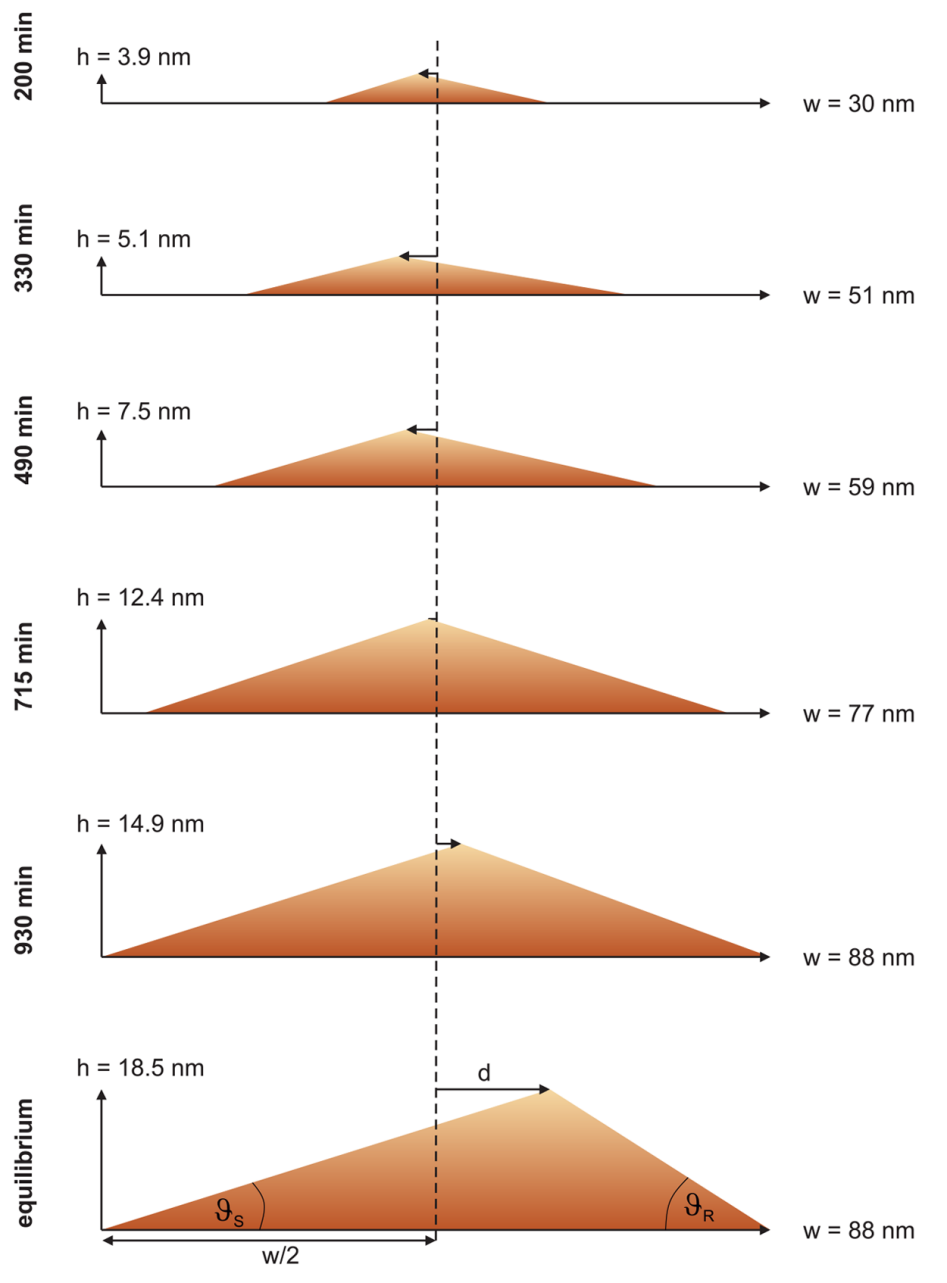
Sketch of the changes in the average ripple profile during surface faceting, with the annealing duration increasing from top to bottom.

With regard to the temporal evolution of the facet inclinations, the curved complex S-plane surface reached its equilibrium considerably faster than the straight simple R-plane surface, as described above. This may be understood by the following consideration: The faceting reconstruction of the sample surface proceeds via mass transport, requiring the release of adatoms from the crystal lattice and their diffusion on the surface. Adatoms can be released from step edges and kinks at lower energetic cost than from lattice positions enclosed in flat surfaces. The curved complex surface contains many such steps and kinks. Thus, mass transport would be more effective on the complex surface than across the flat simple surface, so that the equilibrium inclination can be reached more quickly on the complex surface. Caused by this difference in the temporal evolution of the S-plane and R-plane facets, the ripple profile changes its asymmetry during the surface reconstruction, as sketched in Figure 6. While initially the R-plane facets cover most of the surface area, their area fraction decreases below 50% as their inclination angle ϑ_*R*_ increases. With time, the ripples therefore change from a left-leaning to a right-leaning profile with the crossover occurring after about *t* = 775 min of annealing.

**Figure 7:**
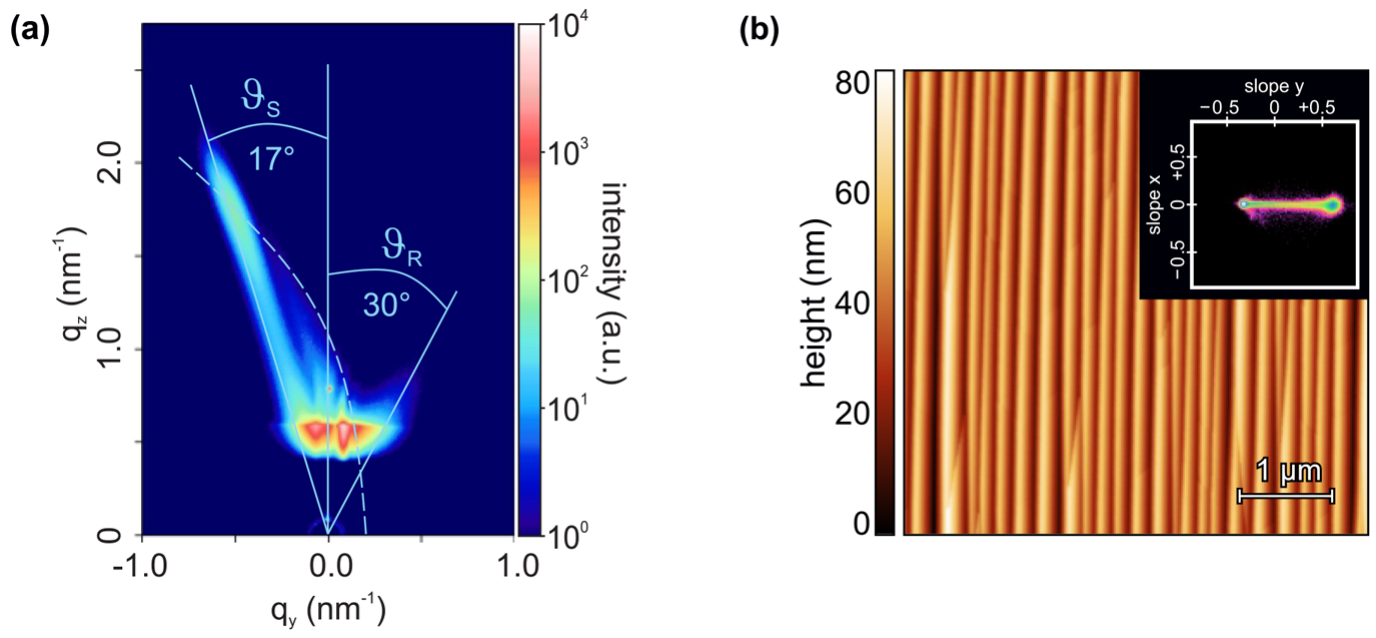
(a) GISAXS intensity map of a α-Al_2_O_3_ sample annealed at 1420 °C for 1380 min. (b) AFM topography image of a reconstructed α-Al_2_O_3_ surface in equilibrium after 2880 min of annealing at 1600 °C. The inset shows the two-dimensional slope histogram of the surface.

We have observed reconstructed surfaces of α-Al_2_O_3_ close to the final equilibrium state in other samples: Figure 7a shows a GISAXS intensity map of a sample annealed at 1420 °C for 1380 min. The AFM data in Figure 7b were taken from a sample annealed at 1600 °C for 2880 min. In both cases the facet inclination angles are very close to their equilibrium values; further approaching the equilibrium values would require unfeasibly long annealing durations. In comparison to Figure 2g, larger ripple width and height as well as a decreased number of ripple junctions can be observed in Figure 7b, which is attributed to enhanced diffusion at higher temperatures.

## Conclusions

We employed *in situ* GISAXS to resolve the surface reconstruction of M-plane α-Al_2_O_3_ into a faceted ripple topography during high-temperature annealing at 1325 °C. Using GISAXS intensity maps calculated for a model surface, we were able to identify the contributions of different surface features to the GISAXS signal. The *in situ* GISAXS data then allowed us to quantify the changes in the ripple dimensions with time: We empirically identified an Johnson–Mehl–Avrami–Kolmogorov type of kinetics for the pattern wavelength and approximately linear increases with time for ripple height, length, and facet angles for most of the observable time span. Thus, the combinatory approach comprising *in situ* GISAXS, *ex situ* AFM, and GISAXS modeling provided an accessible and comprehensive picture of the nanoscale structure formation process. Future experiments employing the latest generation of synchrotrons and area detectors enabling increased sensitivity and time resolution could provide more insights into the very early stages of pattern formation with weak off-specular scattering. *In situ* X-ray photon correlation spectroscopy (XPCS) could further elucidate the different diffusion dynamics on the two ripple surfaces. Thereby, the complete surface reconstruction process would be observable in great detail and in real time. Our proof-of-principle experiments were limited by the challenging alignment during thermal expansion in a high-temperature setup but showcased the utility of modeling GISAXS data and the wealth of information to be gained from *in situ* GISAXS even under such extreme conditions. The observed kinetics of temperature-induced nanopatterning in M-plane α-Al_2_O_3_ will help selecting the appropriate annealing conditions for obtaining the desired facet profiles for specific applications in nanofabrication.

## Supporting Information Available

Sketches and photographs of the *in situ* GISAXS setup (pages S-2f); list of model parameters (pages S-4ff); comparison of calculated and experimental GISAXS intensity maps for illustrating the effect of azimuthal sample rotation (page S-9); comparison of fits to the time dependence of the pattern period λ_*y*_ (pages S-10f) (PDF)
Video of experimental GISAXS intensity data for azimuthal sample rotation from −15° to +15°, showing the bent grating truncation rod and the sensitive dependence of the scattering pattern on azimuthal sample rotation; these data were recorded at the beamline P03 of the PETRA-III synchrotron, at an X-ray energy of 13 keV, a sample-to-detector distance of 3.47 m, an incidence angle of 0.5°, a rotation step width of 0.25°, and an exposure time of 0.5 s per frame (MP4)
