# Supplementary material for: Real-Time Observation of Temperature-Induced Surface Nanofaceting in M-Plane α-Al2O3
Source: ACS Appl Mater Interfaces. 2022 Jun 28;14(27):31373–84. doi: 10.1021/acsami.1c22029 (PMC9284515; doi:10.1021/acsami.1c22029)
Supplement: Supplementary file 1 — am1c22029_si_002.pdf [file am1c22029_si_002.pdf]

Supporting Information

„Real-time observation of temperature-induced surface nanofaceting in M-plane α-Al_2O_3"

Denise J. Erb*[1,2], Jan Perlich[2,3], Stephan V. Roth[2], Ralf Röhlsberger[2,4,5,6], Kai Schlage[2]

1 Institute of Ion Beam Physics and Materials Research, Helmholtz-Zentrum Dresden-Rossendorf HZDR, 01328 Dresden, Germany
2 Photon Science Department, Deutsches Elektronen-Synchrotron DESY, 22607 Hamburg, Germany
3 present address: Continental Reifen Deutschland GmbH, 30419 Hanover, Germany
4 Institut für Optik und Quantenelektronik, Friedrich-Schiller-Universität Jena, 07743 Jena, Germany
5 Helmholtz Institute Jena, 07743 Jena, Germany
6 Helmholtz Centre for Heavy Ion Research GSI, 64291 Darmstadt, Germany
* E-mail: d.erb@hzdr.de

pages S-2f.: Sketches and photographs of the *in-situ* GISAXS setup

pages S-4ff.: List of model parameters

page S-9: Influence of azimuthal sample rotation on GISAXS intensity maps

pages S-10f.: Comparison of fits to the time dependence of the pattern wavelength λ_y

page S-12: Introduction ot supplementary video

in situ GISAXS experimental setup

For in-situ GISAXS during high-temperature annealing, a tube furnace was set up at the beamline BW4 of the DORIS-III synchrotron such that the X-ray beam could pass through the tube in grazing incidence geometry, as sketched in Fig. S1(a). A two axis goniometer allowed for compensating any sample tilt perpendicular to the X-ray incidence direction and for setting the polar angle of incidence $\alpha_i$of the X-rays. A flight tube was installed to evacuate the sample-to-detector distance of 1840 mm and thus reduce scattering in air, see Fig. S1(b). The flight tube window entrance window and the primary beam exit window were shielded from the heat emitted by the furnace by means of Al-coated mylar foils. The beam size was 400 µm x 400 µm. A sample was placed onto a custom-made ceramic support at the edge of the furnace tube and then the support was pushed into the middle of the tube, see Fig. S1(c-d). The ceramic support held the sample in the center of the furnace tube and aligned the sample edges in [1-210] orientation approximately parallel to the azimuthal direction of the incident X-ray beam. Viewing the sample along the incident X-ray beam direction, the facets with R-plane (S-plane) orientation were on the right (left) hand side of each ripple.

in situ GISAXS experimental setup

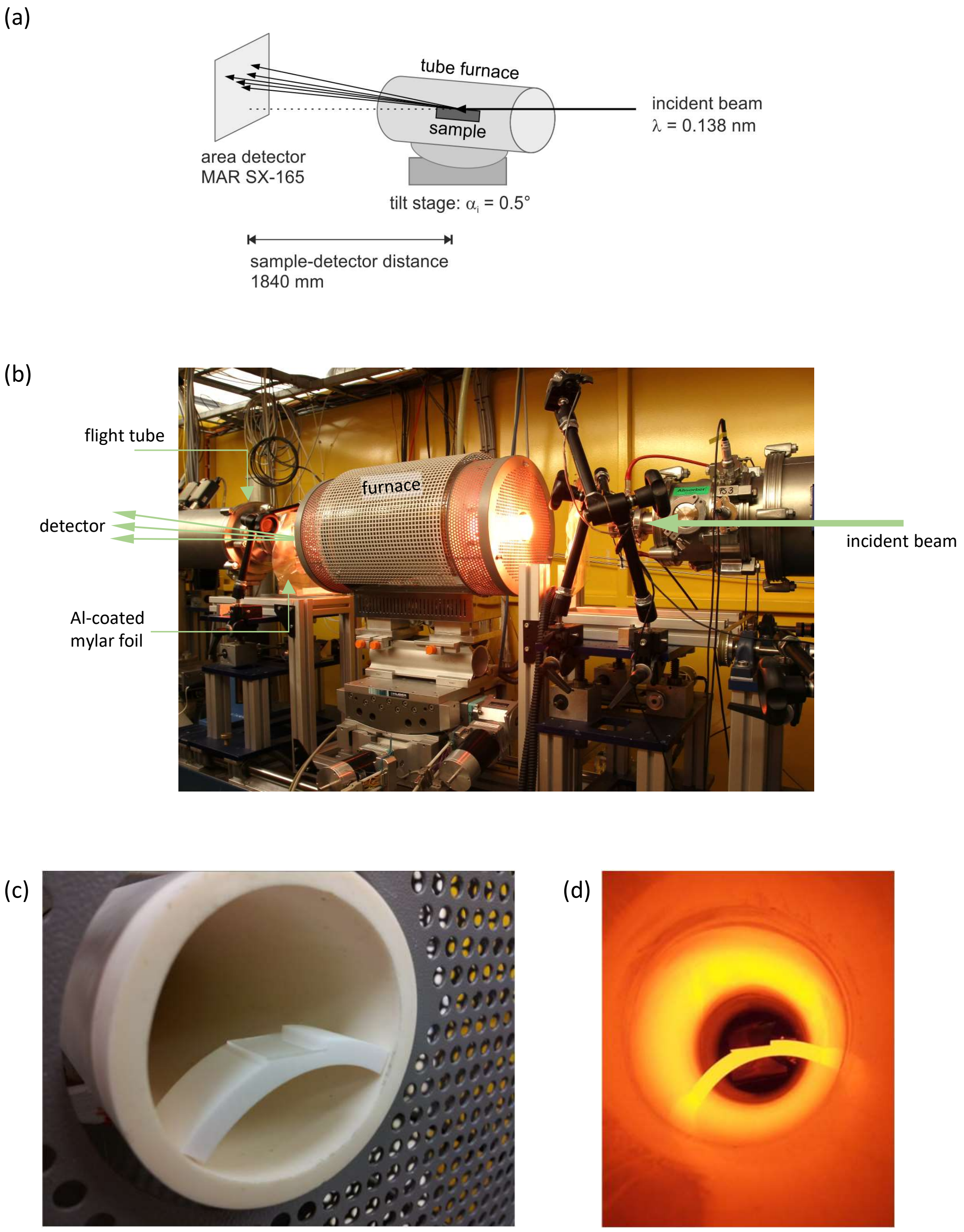

Figure S1:
(a) sketch of the experimental setup; photographs of
(b) the tube furnace at beamline BW4 during the experiment
(c) the sample on the custom-made sample holder at the furnace tube entrance before the experiment
(d) the sample on the holder inside the furnace tube during the experiment

List of model parameters

For modelling the GISAXS intensity maps we used version v1.15.0 (2019-02-25) of the BornAgain software (see Pospelov et al., J. Appl. Cryst. 53, 2020, 262-276). Detailed explanations of all parameters can be found in the software documentation at https://www.bornagainproject.org

List of model parameters

Table S1: Parameters regarding the instrument which were varied to adjust the model to the experimental data. Parameters with errors were fitted; parameters without errors were set. Adjusting the direct beam position v_o relative to the detector accounts for slight changes in sample vertical positioning during the experiment.

**annealing duration**	**instrument parameter**		
	azimuthal angle /°	direct beam v_0 / mm	background intensity / arb. units
170 min	2.70 ± 2.03	-10.5 ± 7.8	1
190 min	1.55 ± 0.89	-11.4 ± 8.6	1
200 min	1.77 ± 1.10	-11.4 ± 3.8	1
250 min	2.47 ± 1.88	-12.1 ± 1.9	1
330 min	2.25	-12.3 ± 1.1	1
410 min	2.14 ± 1.45	-12.3 ± 0.9	2
490 min	2.28 ± 0.84	-12.2 ± 1.2	1
595 min	2.22 ± 0.72	-12.1 ± 0.7	2
715 min	2.2	-12.3 ± 0.4	2
835 min	2.2	-12.3 ± 0.2	2
930 min	2.15	-12.4 ± 0.5	2

List of model parameters

Table S2: Parameters regarding the interference function which were varied to adjust the model to the experimental data. Parameters with errors were fitted; parameters without errors were set.

**annealing duration**	**interference function parameter**			
	position variance / nm	peak distance / nm	HW of probability distribut. / nm	particle density / $10^{-5}nm^{-2}$
170 min	12.5	15 ± 10	2.5	75
190 min	25	41 ± 22	10	4.5
200 min	50	55	10	2.0
250 min	25	66 ± 22	12.5	5.3 ± 1.8
330 min	15	77 ± 11	15	5.1 ± 1.3
410 min	10	77 ± 10	15	5.0 ± 1.4
490 min	10	75 ± 10	15	5.1 ± 1.3
595 min	5	76 ± 7	15	4.5 ± 1.3
715 min	5	74 ± 8	15	3.5 ± 0.5
835 min	5	75 ± 9	15	2.9 ± 0.4
930 min	5	75 ± 9	15	3.0 ± 0.4

List of model parameters

Table S3: Parameters regarding the particle geometry which were varied to adjust the model to the experimental data. Parameters with errors were fitted; parameters without errors were set. Rotating the particles 180° in plane is necessary for the last two cases, because the BornAgain software does not accept asymmetry lengths d < 0.

**annealing duration**	**particle parameter**				
	length l/nm	width w/nm	height h/nm	asymmetry length d/nm	Z rotation /°
170 min	150 ± 104	9 ± 6	2.6	1	0
190 min	450 ± 96	15	3.8 ± 2.4	1	0
200 min	455	30	3.9 ± 1.3	2.5	0
250 min	464 ± 138	43 ± 15	4.3 ± 1.1	4.5	0
330 min	516 ± 102	51 ± 11	5.1 ± 1.0	5	0
410 min	521 ± 107	54 ± 11	6.5	4.5	0
490 min	516 ± 112	59 ± 13	7.5	4	0
595 min	550	70	10	3	0
715 min	587 ± 77	77 ± 9	12.4 ± 1.3	1	0
835 min	622 ± 75	86 ± 8	13.8 ± 1.0	1	180
930 min	633 ± 72	88 ± 7	14.9 ± 1.3	3	180

List of model parameters

Table S4: Settings for the χ^2 minimization used for fitting the model GISAXS intensity maps to the experimental data.

parameter	setting
Minimizer	Minuit2
Algorithms	Migrad
Minimization strategy	1 – medium quality
Error definition factor	1.000
Tolerance	0.010
Arithmetic precision	-1.000
Max. number of function calls	0
Intensity function	none
Variance	Simulation value based
epsilon	1.000

Influence of azimuthal sample rotation on GISAXS intensity maps

The following figure shows calculated and experimental GISAXS intensity maps representing successive stages of surface reconstruction from left to right. The comparison demonstrated the influence of azimuthal sample rotation.

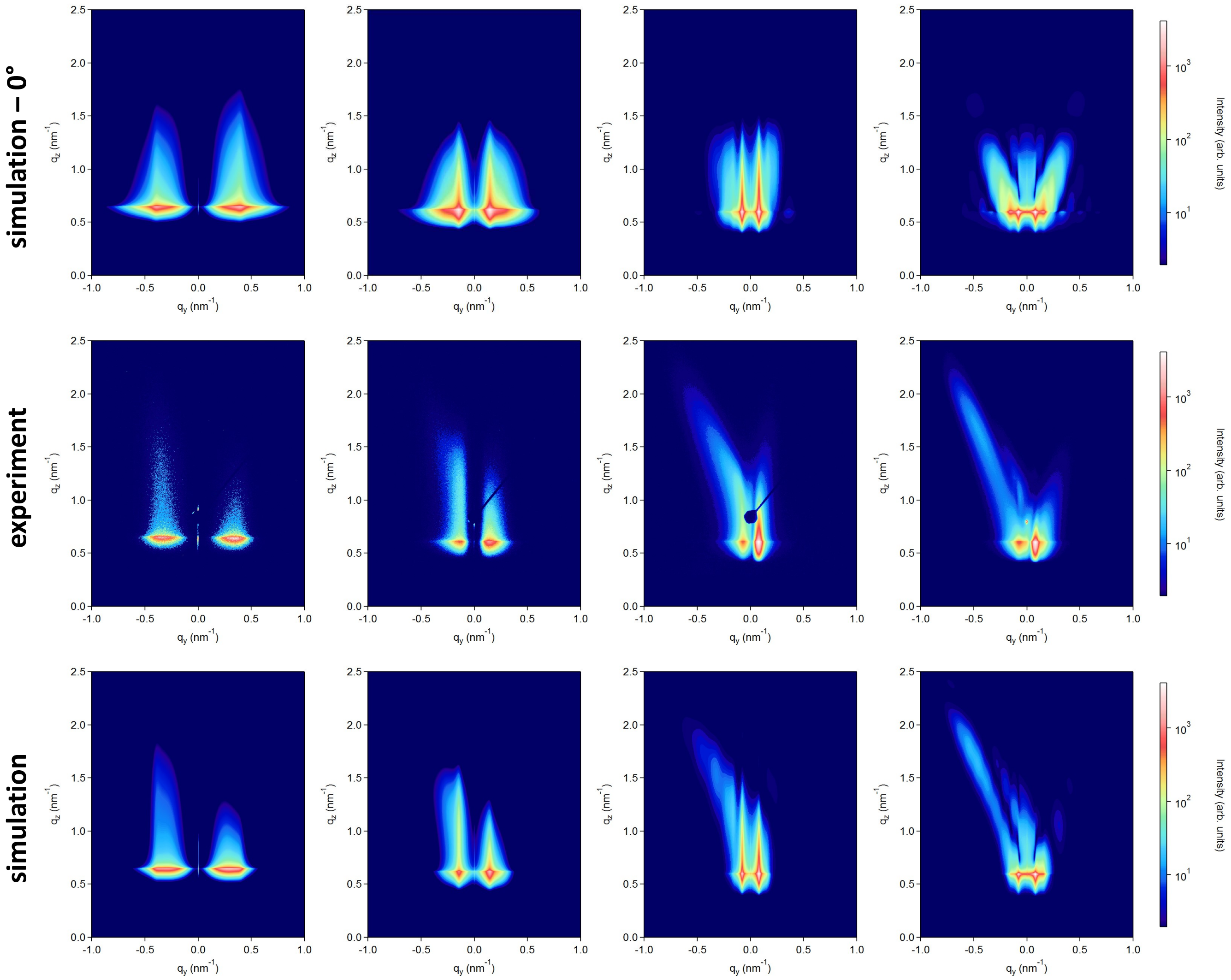

Figure S2:
(top) model, without azimuthal rotation of the sample
(middle) experimental data
(bottom) model, including azimuthal rotation of the sample
The modeled data used parameters as given on pages S-3ff., except for the azimuthal sample rotation.

Comparison of fits to the time dependence of the pattern wavelength λ_y

We find that the observed time-dependence of the pattern wavelength can be described well by a Johnson-Mehl-Avrami-Kolmogorov type of function (see following page). This equation was derived for describing the transformed fraction with time in diffusion-controlled structural transformations of homogeneous systems subject to isothermal annealing. While a JMAK type of equation does fit our data and comparable data of another study (Heffelfinger et al., Surface Science 389 (1997)) better than a power law fit, we do not claim that the surface reconstruction of Al_2O_3 is in fact a transformation process such as defined above. The physical reasons explaining why a JMAK type of equation is a suitable description of the time-dependence of the pattern wavelength in Al_2O_3 reconstruction remain to be clarified.

Comparison of fits to the time dependence of the pattern wavelength λ_y

JMAK equation: $$\lambda_y(t) = \lambda_{y,0}\left(1 - e^{-(kt)^n}\right) \qquad \text{(Eq. S1)}$$

Power law: $$\lambda_y(t) = a(t - t_0)^{1/b} \qquad \text{(Eq. S2)}$$

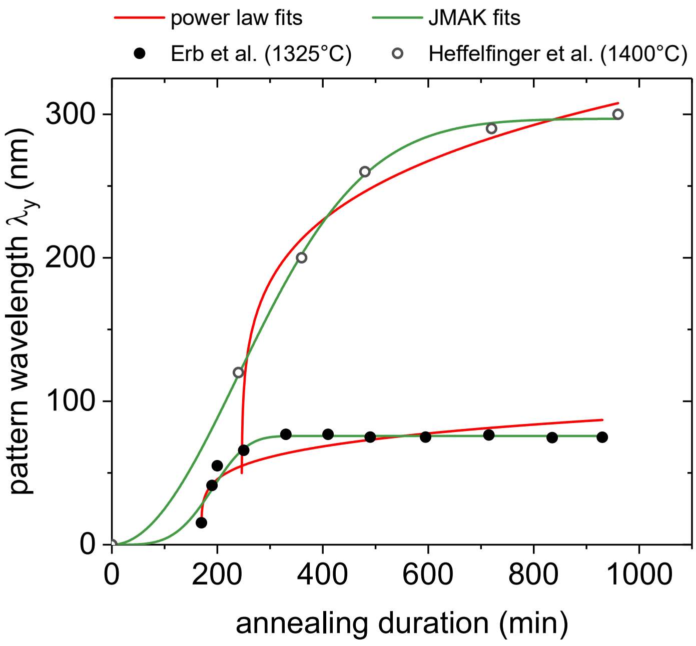

Figure S3:
Comparison of experimental data of the temporal evolution of the pattern wavelength with fits according to a power law and to the Johnson-Mehl-Avrami-Kolmogorov (JMAK) type equation stated above.

**fit**	**R^2**	**a**	**b**	**t_0**	**k**	**n**	**λ_0**
Heffelfinger et al. – power law	0.972	82.7 ± 1.9	5	246.6 ± 3.6			
Heffelfinger et al. – JMAK	0.9995				0.00297 ± 3.7e-5	2	297.0 ± 2.2
Erb et al. – power law	0.819	23.1 ± 0.9	5	169.9 ± 0.5			
Erb et al. – JMAK	0.968				0.00484 ± 1.4e-4	4	75.8 ± 1.9

Table S5:
Parameters of the fits plotted in Fig. S3.
Values without errors were fixed for the fit.

Introduction to supplementary video

The file “SupportingInformation.avi” contains a video of experimental GISAXS intensity data for azimuthal sample rotation from -15° to +15°, showing the bent grating truncation rod and the sensitive dependence of the scattering pattern on azimuthal sample rotation. These data were recorded at the beamline P03 of the PETRA-III synchrotron, at an X-ray energy of 13 keV, a sample-to-detector distance of 3.47 m, an incidence angle of 0.5°, a rotation step width of 0.25°, and an exposure time of 0.5s per frame. The sample had been subject to high temperature annealing beforehand and was at room temperature during the data acquisition.
